# Research Progress on Chemical Constituents and Anticancer Pharmacological Activities of *Euphorbia lunulata* Bunge

**DOI:** 10.1155/2020/3618941

**Published:** 2020-01-14

**Authors:** Yuwei Wang, Xiao Yu, Lingna Wang, Fang Zhang, Yongqing Zhang

**Affiliations:** ^1^College of Pharmacy, Shandong University of Traditional Chinese Medicine, Jinan 250355, China; ^2^Shandong Medicine Technician College, Tai'an 271016, China

## Abstract

*Euphorbia lunulata* Bunge (ELB) is a traditional Chinese medicine possessing the functions of expectoration, cough relief, asthma relief, detoxification, and itching relief. Modern pharmacological studies have showed that ELB exhibits a variety of activities, such as antitumor, antibacterial, and antioxidant activities. In particular, the anticancer activities of ELB have attracted much attention. In this review, we summarize the recent research progress on the chemical constituents and anticancer activities of ELB by searching the PubMed, Web of Science, and China National Knowledge Infrastructure databases. The results show that more than 151 components have been identified from extracts of ELB, including 73 terpenoids, 28 flavonoids, 8 phenylpropanoids, 7 steroids, 19 phenols, and 5 alkaloids. ELB has been shown to exhibit significant inhibitory effects on lung, cervical, gastric, breast, and liver cancers, and its anticancer effects are mainly manifested in the 3 aspects, including cell cycle arrest, cell apoptosis, and inhibition of the migration of cancer cells.

## 1. Introduction

Cancer, one of the most common malignancy tumors, is a group of diseases characterized by irregular unlimited growth and proliferation of abnormal cells, as well as imbalance of apoptosis [1]. In 2012, the WHO's GLOBOCAN estimated approximately 14.1 million new cancer cases and 8.2 million deaths worldwide, which are expected to rise to 22 million in the next two decades [2]. Current methods for treating cancers, such as chemotherapy, radiotherapy, molecular targeted therapy, and immunotherapy, are not always satisfactory. Among them, molecular targeted therapy and radiotherapy have significant side effects on patients, especially elderly patients. Furthermore, chemotherapy drugs typically have a single selectivity and low tolerance [3–5]. Therefore, finding and developing more effective and safer methods for treating cancers have become an important research direction for researchers.

Research into the sources of new drugs indicates that natural products play an important role in drug discovery and development for the treatment of some human diseases, including for tumors. According to incomplete statistics, anticancer agents derived from botanical drugs accounted for 32.25% of total anticancer drugs [6, 7]. In addition, some ingredients extracted from natural products (e.g., camptothecin, taxol, vinblastine, and vincristine) have become the first choice for treating some tumors [8–11]. China is abundant in traditional Chinese medicine (TCM) resources. Chinese herbal medicines have the advantages of rich active ingredients, diverse structures, limited side effects, and a wide anticancer spectrum; therefore, the extraction of new antitumor active compounds from TCM has become a widely popular topic in recent years [12–18].

*Euphorbia lunulata* Bunge (ELB), recorded as *Euphorbia esula* L. in *Chinese flora* [19], is a traditional Chinese medicine, mainly distributed in the northeastern and northern regions of China, which is widely distributed in Eurasia and naturalized in North America and worldwide [19]. ELB possessed the functions of expectoration, cough relief, asthma relief, detoxification, and itching relief and has been widely used in the treatment of phlegm, coughs, asthma, edema, scabies, and anonymous swelling [20], as well as for lung cancer, gastric cancer, lymph node nucleus [21], rheumatic immune diseases [22], and psoriasis [23]. Modern pharmacological studies have shown that ELB exhibits a variety of activities, such as antitumor, antibacterial [24], and antioxidant [25] activities. Studies have shown that ELB has significant anticancer activity, whether used alone [26] or in combination with other drugs [27].

In recent years, scholars, both at home and abroad, have showed great interest in ELB because of its anticancer efficacy. Thus, many of the chemical constituents of ELB have been isolated, such as terpenoids, flavonoids, steroids, phenylpropanoids, phenols, and other chemical constituents [28–53]; yet, few of these ingredients have been tested for their anticancer activities. Until recently, not much attention has been paid to the screening of its active anticancer components. Therefore, a comprehensive and systemic review of the chemical constituents of ELB and their associated anticancer activities is indispensable for its further research and clinical applications.

## 2. Constituents

More than 151 compounds have been isolated and identified from ELB so far, including diterpenoids, triterpenes, flavonoids, steroids, alkaloids, coumarins, lignins, phenols, and volatile oils.

### 2.1. Terpenoids

The terpenoids have been given more attention since the discovery of taxol, and the terpenoids are the most abundant components in ELB. Up to now, 73 terpenoids have been isolated and identified from ELB, which contained 56 diterpenoids and 17 triterpenoids.

#### 2.1.1. Diterpenoids

Euphorbia is rich in diterpenoids. So far, there have been more than 56 kinds of diterpenoids isolated from ELB, which can be classified into four classes: ingenane-type, jatrophane-type, abietane-type, and other types of diterpenes.

**Figure 1:**
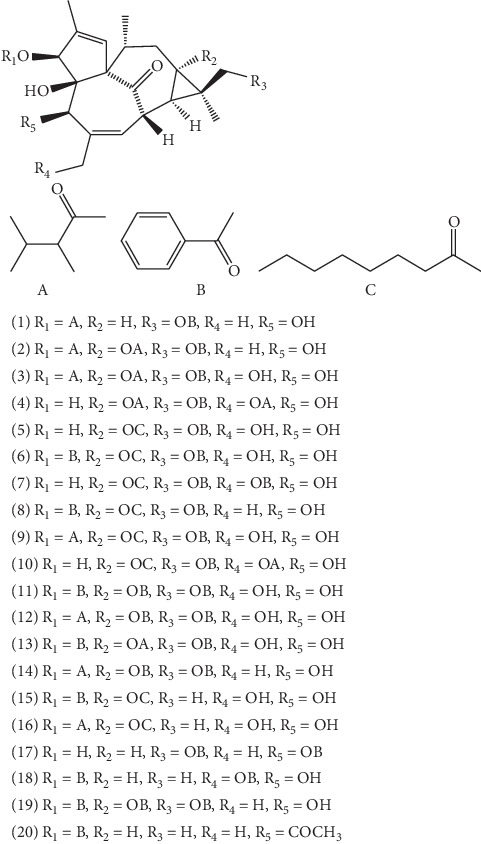
The structures of 20 ingenane-type diterpenoids (**1**–**20**).

Among them, 20 kinds of ingenane-type diterpenoids (see Figure 1) have been isolated from ELB, including 17-benzoyloxy-3-O-(2,3-dimethylbutanoyl)-20-deoxyingenol **(1)** [28], 17-benzoyloxy-3-O-(2,3-dimethylbutanoyl)-13-(2,3-dimethylbutanoyloxy)-20-deoxyingenol **(2)** [28], 7-benzoyloxy-3-O-(2,3-dimethylbutanoyl)-13-(2,3-dimethylbutanoyloxy)ingenol **(3)** [28], 7-benzoyloxy-20-O-(2,3-dimethylbutanoyl)-13-(2,3-dimethylbutanoyloxy)ingenol **(4)** [28], 17-benzoyloxy-13-octanoyloxyingenol **(5)** [28], 3-O-benzoyl-17-benzoyloxy-13-octanoyloxyingenol **(6)** [28], 20-O-benzoyl-17-benzoyloxy-13-octanoyloxyingenol **(7)** [28], 3-O-benzoyl-17-benzoyloxy-13-octanoyloxy-20-deoxyingenol **(8)** [28], 17-benzoyloxy-3-O-(2,3-dimethylbutanoyl)-13-octanoyloxyingenol **(9)** [28], 17-benzoyloxy-20-O-(2,3-dimethylbutanoyl)-13-octanoyloxyingenol **(10)** [28], 3-O-benzoyl-13,17-dibenzoyloxyingenol **(11)** [28], 13,17-dibenzoyloxy-3-O-(2,3-dimethylbutanoyl)ingenol **(12)** [28], 3-O-benzoyl-17-benzoyloxy-13-(2,3-dimethylbutanoyloxy)ingenol **(13)** [28], 13,17-dibenzoyloxy-3-O-(2,3-dimethylbutanoyl)-20-deoxyingenol **(14)** [28], 3-O-benzoyl-13-octanoyloxyingenol **(15)** [28], 3-O-(2,3-dimethylbutanoyl)-13-octanoyloxyingenol **(16)** [28], 6-benzoyloxy-20-deoxyingenol-5-benzoate **(17**) [29], ingenol-3,20-dibenzoate **(18)** [30], 13,16-dibenzoyloxy-20-deoxyingeno-3-benzoate **(19)** [31], and kansuiphorin D **(20)** [32].

**Figure 2:**
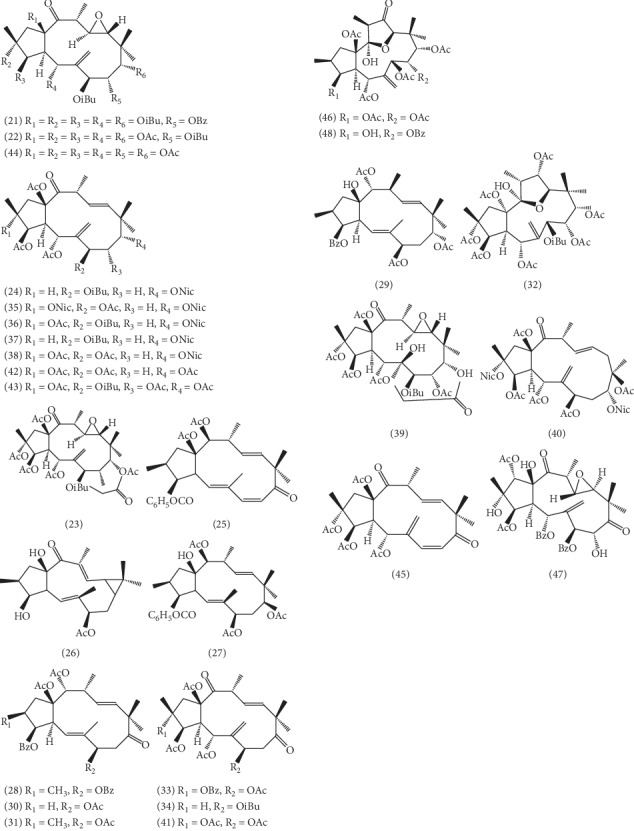
The structures of 28 jatrophane-type diterpenoids (**21**–**48**).

As shown in Figure 2, 28 kinds of jatrophane-type diterpenoids have been isolated from ELB, such as 2*α*,3*β*,5*α*,9*α*,15*β*-pentaacetoxy-11,12-epoxy-7*β*-isobutyryl-8*α*-benzoyloxyjatropha-6(17)-en-14-one **(21)** [33], salicinolide **(22)** [34], 2*α*,3*β*,5*α*,9*α*,15*β*-pentaacetoxy-11,12-epoxy-7*β*,8*α*-diisobutyryloxyjatropha-6(17)-en-14-one **(23)** [34], 3*β*,5*α*,15*β*-triacetoxy-7*β*-isobutyryloxy-9*α*-nicotinoyloxyjatropha-6(17),11(E)-dien-14-one **(24)** [34], euphoscopin M **(25)** [32], alisol A **(26)** [32], euphornin A **(27)** [32], 14*α*,15*β*-diacetoxy-3*α*,7*β*-dibenzoyloxy-9-oxo-2*β*H,13-*α*Hjatropha-5E,11E-diene **(28)** [35], euphornin **(29)** [35], euphoscopin B **(30)** [35], euphornin N **(31)** [35], esulatinsH(2R,3*β*,5R,8R,9R,12R,15*β*-heptaacetoxy-11,14-epoxy-14R-hydroxy-7*β*-isobutanoyloxyjatropha-6(17)-ene) **(32)** [36], esulatinsI(3,5,7,15-tetraacetoxy-2-benzoyloxyjatropha-6(17),11-diene-9,14-dione) **(33)** [36], esulatinsJ(3*β*,5R,15*β*-triacetoxy-7*β*-isobutanoyloxyjatropha-6(17),11Ediene-9,14-dione) **(34)** [36], esulatinsK **(35)** [36], esulatinsL(2R,3*β*,5R,15*β*-tetraacetoxy-7*β*-isobutanoyloxy-9R-nicotinoyloxyjatropha-6(17),11E-dien-14-one) **(36)** [36], esulatinsM(3*β*,5R,15*β*-triacetoxy-7*β*-isobutanoyloxy-9R-nicotinoyloxyjatropha-6(17),11-dien-14-one) **(37)** [36], 2R,3*β*,5R,7*β*,15*β*-pentaacetoxy-9R-nicotinoyloxyjatropha-6(17),11-dien-14-one **(38)** [36], salicinolide **(39)** [36], euphosalicin **(40)** [36], esulatinsA(2R,3R,4S,5R,7S,8R,9S,11R,12S,13R,15R)-2,3,5,8,9,15hexaacetoxy-11,12-epoxy-7-(isobutanoyloxy)jatroph-6(17)en-14-one **(41)** [37], esulatinsB(2,3,5,7,15-pentaacetoxyjatropha-6(17),11-diene-9,14-dione) **(42)** [37], esulatinsC **(43)** [37], esulatinsD **(44)** [38], esulatins (2,3,5,15-tetraacetoxyjatropha-6(17),7E,11E-triene-9,14-dione) **(45)** [38], 11,14-epoxy-3*β*,5*α*,7*β*,8*α*,9*α*,15*β*-hexaacetoxy-12-oxo-13*α*H-jatropha-6(17)-ene **(46)** [39], 1*α*,3*β*-diacetoxy-5*α*,7*β*-dibenzoyloxy-9,14-dioxo-11*β*,12*α*-expoxy-2*α*,8*α*,15*β*-trihydroxy-13*α*H-jatropha-6(17)-ene **(47)** [39], and esulol A **(48)** [40].

**Figure 3:**
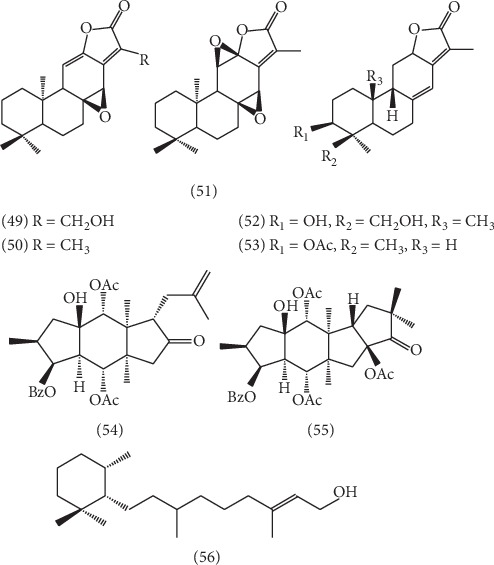
The structures of 5 abietane type and 3 other types of diterpenoids (**49**–**56**).

5 kinds of abietane-type diterpenoids, including 17-hxdroxyjolkinolide A **(49)** [41], jolkinolide A **(50)** [41], jolkinolide B **(51)** [41], 18-hydroxyhelioscopinolide A **(52)** [32], and Ent-3*α*-formylabieta-8(14),13(15)-dien-16,12*β*-olide **(53)** [35], have been isolated from ELB; in addition, 3 other types of diterpenes including esulatinG **(54)** [42], 5,8,14-triacetoxy-3-benzoyloxy-15-hydroxy-9-oxo-paraliane **(55)** [42], and cassipourol **(56)** [34] have been isolated; the structures of these 8 compounds are shown in Figure 3.

#### 2.1.2. Triterpenes

**Figure 4:**
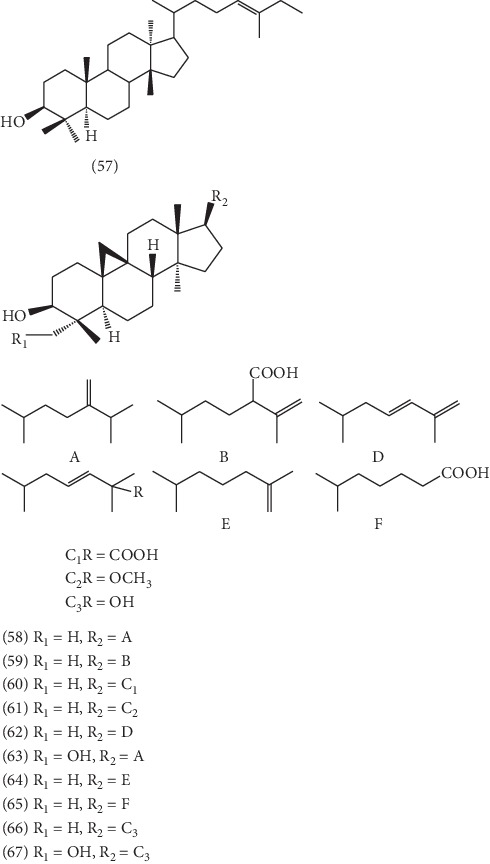
The structures of 11 tetracyclic triterpenoids **57**–**67**.

**Figure 5:**
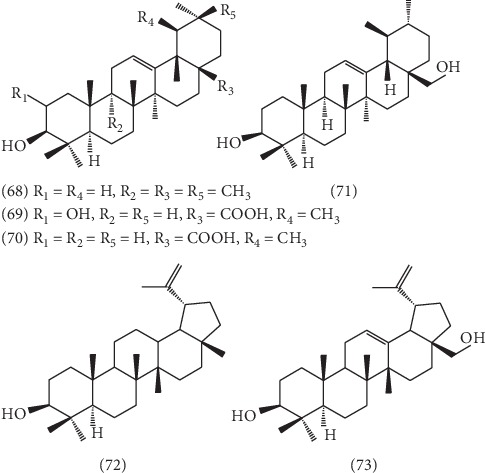
The structures of 6 pentacyclic triterpenoids **68**–**73**.

So far, 17 triterpenoids were isolated from the ELB, which could be classified into tetracyclic triterpenes and pentacyclic triterpenes. Among them, except for euphor **(57)** [34], the other 10 tetracyclic triterpenes (Figure 4) were all cycloartane-type triterpenes, including 24-methylenecycloartan-3*β*-ol **(58)** [43], 25-hydroperoxycycloart-23-en-3*β*-ol **(59)** [34], 25-hydroperoxycycloart-23-en-3*β*-ol **(60)** [34], (23E)-25-methoxycycloart-23-en-3*β*-ol **(61)** [34], (23E)-cycloart-23,25-dien-3*β*-ol **(62)** [34], 24-methylenecycloartan-3*β*,28-diol **(63)** [34], 3-hydroxyl-cycloartane-25-ene **(64)** [34], 25,26,27-trinor-3*α*-hydroxycycloartan-24-oic acid **(65)** [35], cycloart-23-ene-3*β*,25-diol **(66)** [35], and cycloart-23-ene-3*β*,25,28-triol **(67)** [35]. And 6 pentacyclic triterpenes including three oleanane-type triterpenoids **(68-70)**, one ursane-type triterpenoid **(71)**, and two lupane-type triterpenoids **(72,73)**, respectively, named *β*-amyrin **(68)** [43], corosolic acid **(69)** [45], ursolic acid **(70)** [35], uvaol **(71)** [34], lupeol **(72)** [34], and betulin **(73)** [34], have been isolated and identified. And the structure of 6 pentacyclic triterpenes is presented in Figure 5.

### 2.2. Flavonoids

**Figure 6:**
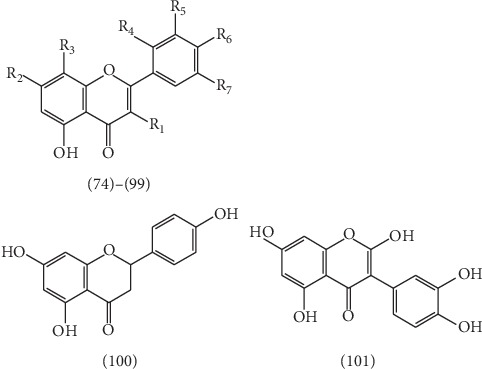
The structures of 28 flavonoids **74**–**101**.

Until now, more than 28 flavonoids have been isolated from ELB, which could be structurally divided into dihydroflavones, isoflavones, and flavonoids. Among them, 26 flavonoids **(74–99)**, such as 3,5,7-trihydroxy-8-methoxyflavone **(74)** [43], naringenin-7-O-*β*-D-glucoside **(75)** [22], quercetin-3-O-(6″-galloyl)-*β*-D-galactopyranoside **(76)** [22], apigenin-7-O-*β*-D-glucoside **(77)** [22], kaempferol-7-O-*β*-D-glucoside **(78)** [44], quercetin-7-O-*β*-D-glucoside **(79)** [44], quercetin-3-O-*α*-L-rha **(80)** [44], kaempferol-3-O-(6″-galloyl)-*β*-D-glucoside **(81)** [44], kaempferol-3-O-*β*-D-glucoside **(82)** [44], jaceosidin **(83)** [45], myricetin-3-O-(2″,3″-digalloyl)-*β*-D-galactopyranoside **(84)** [46], myricetin-3-O-(2″-galloyl)-*β*-D-galactopyranoside **(85)** [46], myricitrin **(86)** [46], myricetin **(87)** [46], apigenin **(88)** [46], luteolin **(89)** [46], 3-O-methylquercetin **(90)** [46], 5,7,2′,5′-tetrahydroxyflavone **(91)** [46], kaempferol **(92)** [47], quercetin **(93)** [47], quercetin-3-L-rha **(94)** [47], kaempferol-3-L-rha **(95)** [47], quercetin-3-O-(2″,3″-digalloyl)-*β*-D-galactopyranoside **(96)** [48], quercetin-3-O-(2″-galloyl)-*β*-D-galactopyranoside **(97)** [48], hyperin **(98)** [48], and quercetin-3-0-*β*-glucuronic **(99)** [49], have been isolated and identified from ELB. In addition, a dihydroflavone and an isoflavone, named 5,7,4′-trihydroxydihydroflavone **(100)** [45] and isoquercetin **(101)** [46], was isolated and identified. The structures of these 28 flavonoids are presented in Table 1 and Figure 6.

### 2.3. Phenylpropanoid

**Figure 7:**
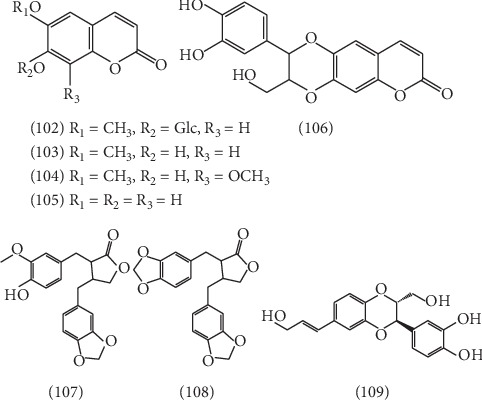
The structures of 8 phenylpropanoids (**102**–**109**).

8 phenylpropanoids **(102–109)** were isolated from ELB, which could be divided into two classes: coumarins and lignans. 5 coumarins have been isolated, including scopolin **(102)** [41], scopoletin **(103)** [22], isofraxidin **(104)** [22], esculetin **(105)** [47], and maoyancaosu **(106)** [47, 50]. 3 lignans were isolated and identified from ELB, respectively, named *trans*-2-(4″-hydroxy-3″-methoxybenzyl)-3-(3′,4′-dimethoxybenzyl)-butyolactone **(107)** [51], *trans*-2-(3″,4″-dimethoxybenzyl)-3-(3′,4′-dimethoxybenzyl)-butyolactone **(108)** [51], and isoanmericanon A **(109)** [51]. The structures of 8 phenylpropanoids are listed in Figure 7.

### 2.4. Steroids

**Figure 8:**
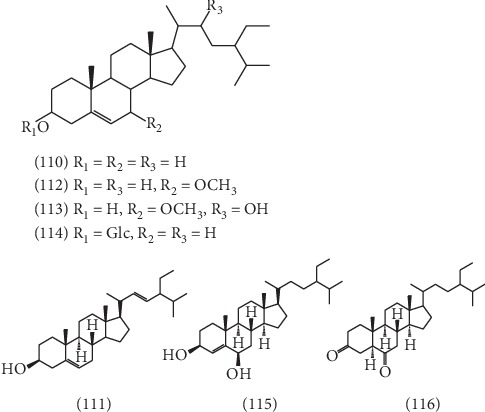
The structures of 7 steroids (**110**–**116**).

As shown in Figure 8, 7 steroids have been isolated from ELB, including *β*-sitosterol **(110)** [44], stigmasterol **(111)** [44], 3*β*-hydroxy-7*β*-methoxy-5-stigmasterone **(112)** [45], 3*β*, 22*β*-dihydroxy-7*β*-methoxy-5-stigmasterone **(113)** [45], *β*-daucosterol **(114)** [32], stigmast-4-ene-3*β*,6*β*-diol **(115)** [35], and stigmastane-3,6-dione **(116)** [35].

### 2.5. Phenols

**Figure 9:**
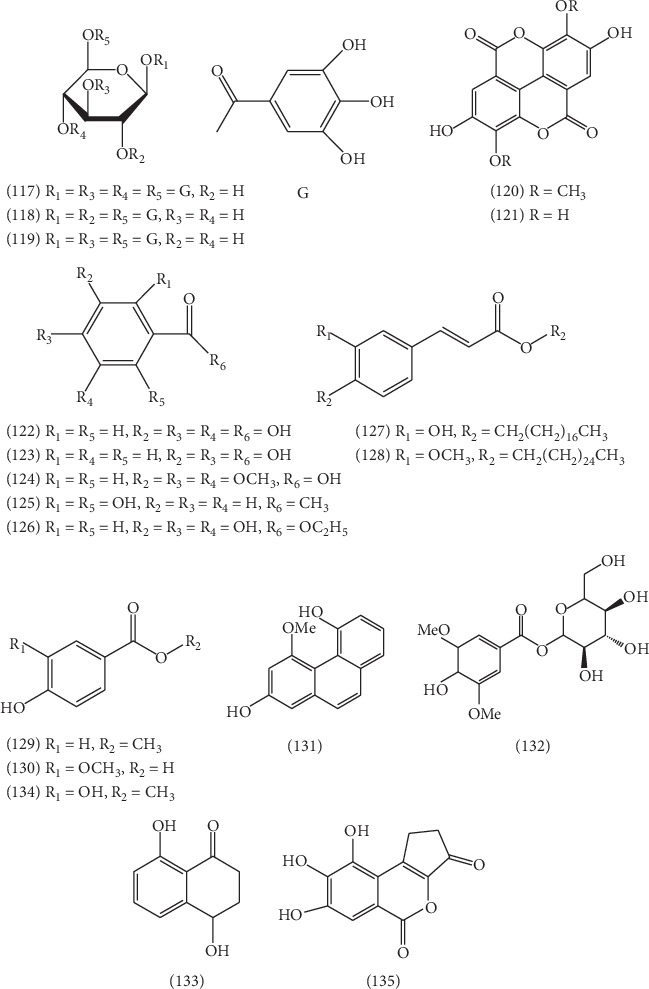
The structures of 19 phenols (**117**–**135**).

19 phenols **(117–135)** have been obtained from ELB, including 1,3,4,6-tetra-O-galloyl-*β*-D-glucose **(117)** [46], 1,2,6-tri-O-galloyl-*β*-D-glucose **(118)** [46], 1,3,6-tri-O-galloyl-*β*-D-glucose **(119)** [46], 3,3′-di-O-methylellagic acid **(120)** [46], ellagic acid **(121)** [46], gallic acid **(122)** [46], protocatechuic acid **(123)** [46], 3,4,5-trimethoxybenzoic acid **(124)** [46], 2,6-dihydroxyacetophenone **(125)** [46], ethyl gallate **(126)** [51], octadecyl caffeate **(127)** [41], hexadecyl ferulate **(128)** [41], *p*-hydroxybenzoic acid **(129)** [45], 3-hydroxy-4-methoxybenzoic acid **(130)** [45], 2,5-dihydroxy-4-methoxy phenanthrene **(131)** [45], erigeside C **(132)** [45], 4,8-dihydroxy-1-tetralone **(133)** [52], methyl 3,4-dihydroxybenzoate **(134)** [52], and vifolin **(135)** [43]. Their structures are listed in Figure 9.

### 2.6. Alkaloids

**Figure 10:**
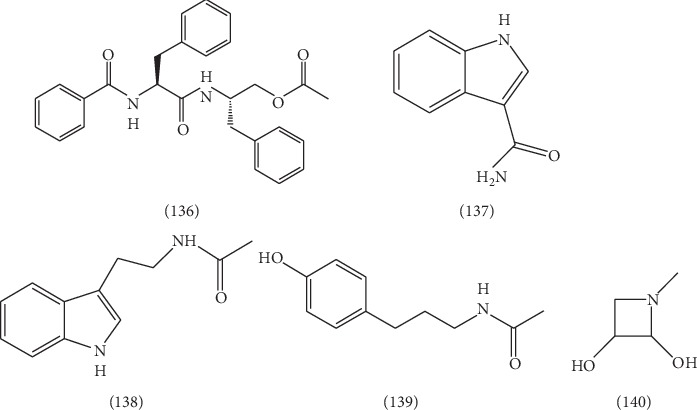
The structures of 5 alkaloids (**136**–**140**).

A few of alkaloids were found from ELB, including asperglaucide **(136)** [45], 1H-3-amido oxime **(137)** [52], *N*-[2-(1H-indol-3-yl)ethyl]acetamide **(138)** [52], *N*-[2-(4-hydroxyphenyl)ethyl]acetamide **(139)** [52], and 2,3-dihydroxy-methyl nitrogen **(140)** [52]. Their structures are presented in Figure 10.

### 2.7. Long-Chain Aliphatic Group

4 compounds, which belonged to the long-chain aliphatic group, were isolated, including *n*-octadecyl alcohol **(141)** [41], cetyl alcohol **(142)** [44], triacontanol **(143)** [44], and tetracatanol **(144)** [44].

### 2.8. Volatile Oils

**Figure 11:**
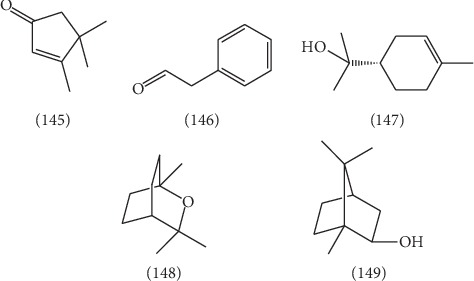
The structures of 5 volatile oils (**145**–**149**).

Wang et al. [53] used steam distillation to extract volatile oil from ELB and analyze the chemical constituents of volatile oil by gas chromatography-mass spectrometry (GC-MS). 43 components were identified, and the relative percentage of each component was calculated by the peak area normalization method. The identified components accounted for 75.86% of the total outflow peak area. Among them, 3 main compounds including 3,4,4-trimethyl-2-cyclopenten-1-one (12.67%) **(145)**, phenylacetaldehyde (12.36%) **(146)**, *α*-terpineol (4.47%) **(147)** and 2 active ingredients including eucalyptol **(148)** and borneol **(149)** were identified. Their structures are shown in Figure 11.

### 2.9. Others

**Figure 12:**
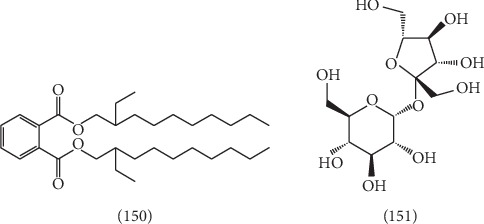
The structures of 2 compounds (**150**–**151**).

Other chemical constituents other than terpenoids, phenylpropanoids, flavonoids, steroids, phenols, alkaloids, long-chain aliphatic group, volatile oils, such as *o*-phthalic acid bis-(2-ethyl decyl)-ester **(150)** [41], and sucrose **(151)** [41], were isolated, the structures of which are shown in Figure 12.

## 3. Antitumor Pharmacological Activity

At present, the mechanism of the occurrence and development of cancer has not been fully revealed; however, it has been confirmed that the proliferation of cancer cells originates from their ability to avoid programmed cell death (i.e., the so-called apoptosis). Therefore, inducing cancer cell apoptosis has been identified as a target for cancer treatment [54–57]. ELB has exhibited antitumor activity, having significant inhibitory effects on lung, cervical, gastric, breast, and liver cancers. Its anticancer effects are mainly manifested in the following aspects: (1) inhibiting the growth of cancer cells by regulating cell cycles, (2) inducing cancer cell apoptosis by regulating the expression of apoptosis-related proteins, and (3) inhibiting the migration of cancer cells by regulating related signaling pathways.

### 3.1. Lung Cancer

Lung cancer is one of the most common malignant tumors, seriously threatening human health. Its incidence and mortality rank high [58].

Xiao et al. and Zhang et al. [59, 60] reported that an aqueous extract of ELB exhibited significant inhibitory activities against the growth of Lewis lung cancer in a mouse model: a flow cytometry assay showed that the S phase percentage of the cell cycle was increased at doses of 30 g·kg^−1^ and 60 g·kg^−1^ with apoptotic rates of (16.43 ± 18.69)% and (24.37 ± 15.48)%, respectively. The apoptotic index (AI) of the 60 g·kg^−1^ group was significantly higher than the control group, with a value of (5.93 ± 5.96)%. Therefore, ELB could induce apoptosis at a dose of 60 g·kg^−1^. Jiang [61] found that a flavonoid extract of ELB could inhibit the growth of Lewis lung cancer mice and that rabbit serum containing ELB could significantly inhibit the proliferation of A549 lung cancer cells in a concentration- and time-dependent manner: at a concentration of 20% for 72 h, the A549 proliferation inhibiting rate was 39.08%; thus, rabbit serum containing ELB extract could induce significant A549 cell apoptosis by arresting the cell in the G1 phase. Western blotting analysis showed that the active ingredients of ELB could inhibit the phosphorylation of EGFR and downregulate AKT and ERK signals. However, Wang et al. [62] found that extracts of ELB could induce the apoptosis of A549 cells, which may be related to an increase in bax expression and a decrease of bcl-2 expression. Liu et al. [35] evaluated in vitro antiproliferative activities against NCI-H460 human lung carcinoma cell lines of five compounds (compounds **28–31** and **53**), where the results showed that compound **53** exhibited marked activity, with an IC50 value of 19.5 *μ*M, and compounds **28**–**31** showed moderate cytotoxic activities, with IC50 values of 58.2 (compound **28**), 53.1 (compound **29**), 33.0 (compound **30**), and 36.7 (compound **31**) *μ*M.

### 3.2. Cervical Cancer

Cervical cancer is the second most common cancer, affecting women's health worldwide [63]. Ming et al. [64] discovered that an ELB extract could efficiently inhibit HeLa cervical cancer cell proliferation and induce the apoptosis of HeLa cells by downregulating the expression levels of Cyclin D1, Cyclin E, and CDK4 proteins and upregulating the expression levels of the P21 protein. Tu et al. [65] reported that an ELB extract could inhibit SiHa human cervical cancer cell proliferation and induce cell apoptosis by increasing the expression levels of caspase-3 and caspase-9.

### 3.3. Gastric Cancer

Cancer multidrug resistance is the term used to describe a phenomenon in which once a cancer develops resistance to one chemotherapeutic drug, it will become resistant to many (or all) other chemotherapeutic drugs (which may or may not have the same mechanism of action). Cancer multidrug resistance has become a problem that cannot be ignored in clinical practice, and many studies are being carried out to find ways to combat or reverse it [66–70]. Fu et al. reported that the proliferation, migration, and invasion of multidrug-resistant human gastric cancer cell line SGC7901/ADR were significantly inhibited by an *n*-hexane extract of ELB in a time- and dose-dependent manner: the cell cycle was arrested in the G2/M phase, and cell apoptosis was induced. The result of the related mechanisms showed that the extract could upregulate the expression of the apoptosis-promoting protein Bax and downregulate that of the apoptosis-inhibiting protein Bcl-2, as well as increasing the activities of caspase-3, caspase-8, and caspase-9 [71, 72]. In addition, the ELB extract could increase the sensitivity of the multidrug-resistant human gastric cancer cell SGC7901/ADR to the chemotherapy drugs paclitaxel and adriamycin, which was manifested in two aspects: it increased the growth inhibitory effects of paclitaxel and adriamycin to SGC7901/ADR gastric cancer cells and significantly reduced the IC50s of paclitaxel and adriamycin to SGC7901/ADR cells, in a concentration-dependent manner [73].

### 3.4. Liver and Breast Cancers

ELB has also exhibited inhibitory effects on the growth of liver and breast cancer cells. Zhang et al. reported that an ethylacetate extract of ELB could inhibit the growth of ZR-75-30 breast cancer cells, where the inhibition rate increased with an increase in drug concentration [74]. Gao et al. [75] found that an extract of ELB inhibited the proliferation of human hepatoma HepG2 cells in a time- and concentration-dependent manner, which may have been related to mitochondrial pathways or cellular apoptosis pathways. Wang et al. [32] isolated four compounds (compounds **20** and **25–27**) which exhibited strong inhibitory activities against HepG2 human hepatocellular carcinoma cells. Liu et al. [35] isolated five compounds (compounds **28–31**,**53**) and evaluated their in vitro antiproliferative activities against MCF-7 breast cancer cells. The results showed that compound **53** exhibited marked activity, with an IC50 value of 18.6 *μ*M, and compounds **28–31** showed moderate cytotoxic activities, with IC50 values ranging from 32.1 to 57.1 *μ*M.

## 4. Discussion and Conclusion

ELB is a traditional Chinese medicine possessing the functions of expectoration, cough relief, asthma relief, detoxification, and itching relief. Modern pharmacological studies have shown that ELB exhibits a variety of activities, such as antitumor, antibacterial, and antioxidant. In particular, its anticancer activities have been paid much attention in recent years. The present review summarizes the chemical constituents and the antitumor activities. The results showed that more than 151 components have been identified from extracts of ELB, including 73 terpenoids, 28 flavonoids, 8 phenylpropanoids, 7 steroids, 19 phenols, and 5 alkaloids. ELB has been shown to exhibit significant inhibitory effects on lung, cervical, gastric, breast, and liver cancers. However, there are limitations associated with most of these studies: (1) most of the studies reported to date have focused exclusively on the use of ELB fractions, as well as the use of its crude extracts; in contrast, there have been very few reports pertaining to the use of single compounds isolated from ELB; (2) most of the studies concerning the anticancer activities and mechanisms have been conducted using in vitro cellular systems, which could explain how nonspecific cell cycle arrest and apoptosis were induced. However, very few reports have focused on specific molecular targets or enzymatic pathways; and (3) to our knowledge, there exist no studies concerned with the anticancer mechanisms of single compounds isolated from ELB. Therefore, systematic research should be carried out to isolate the monomer components and screen the anticancer activity of these components, in order to confirm the anticancer active ingredients and further study on the anticancer activities and mechanisms of these ingredients, which will be useful in discovering new active anticancer single compounds and new anticancer drug resources.

## Figures and Tables

**Table 1:** The structures of 26 flavonoids **74**–**99**.

Comp. number	Substitutional groups							References
	R_1_	R_2_	R_3_	R_4_	R_5_	R_6_	R_7_	
**74**	OH	OH	OCH_3_	H	H	H	H	[43]
**75**	H	O-*β*-D-Glc	H	H	OH	OCH_3_	H	[22]
**76**	O-(6″-Galloyl)-*β*-D-Gal	OH	H	H	OH	OH	H	[22]
**77**	H	O-*β*-D-Glc	H	H	H	OH	H	[22]
**78**	OH	O-*β*-D-Glc	H	H	H	OH	H	[44]
**79**	OH	O-*β*-D-Glc	H	H	OH	OH	H	[44]
**80**	O-*α*-L-Rha	OH	H	H	OH	OH	H	[44]
**81**	O-(6″-O-Galloyl)-*β*-D-Glc	OH	H	H	H	OH	H	[44]
**82**	O-*β*-D-Glc	OH	H	H	H	OH	H	[44]
**83**	H	OH	H	H	H	OCH_3_	H	[45]
**84**	O-(2″,3″-Digalloyl)-*β*-D-Gal	OH	H	H	OH	OH	OH	[46]
**85**	O-(2″-Galloyl)-*β*-D-Gal	OH	H	H	OH	OH	OH	[46]
**86**	O-*α*-L-Rha	OH	H	H	OH	OH	OH	[46]
**87**	OH	OH	H	H	OH	OH	OH	[46]
**88**	H	OH	H	H	H	OH	H	[46]
**89**	H	OH	H	H	OH	OH	H	[46]
**90**	OCH_3_	OH	H	H	OH	OH	H	[46]
**91**	OH	H	H	OH	H	H	OH	[46]
**92**	OH	OH	H	H	H	OH	H	[47]
**93**	OH	OH	H	H	OH	OH	H	[47]
**94**	O-L-Rha	OH	H	H	OH	OH	H	[47]
**95**	O-L-Rha	OH	H	H	H	OH	H	[47]
**96**	O-(2″,3″-Digalloyl)-*β*-D-Gal	OH	H	H	OH	OH	H	[48]
**97**	O-(2″-Galloyl)-*β*-D-Gal	OH	H	H	OH	OH	H	[48]
**98**	O-*β*-D-Gal	OH	H	H	OH	OH	H	[48]
**99**	O-*β*-D-Glc	OH	H	H	OH	OH	H	[49]
